# ﻿Introducing *Muciflexusinthanonensis* gen. et sp. nov. and updates on *Ochronectria* (Hypocreales): New insights from leaf litter fungi in Doi Inthanon National Park, Northern Thailand

**DOI:** 10.3897/mycokeys.117.147002

**Published:** 2025-05-05

**Authors:** Veenavee S. Hittanadurage Silva, Ruvishika S. Jayawardena, Rekhani H. Perera, Qirui Li, Kevin D. Hyde

**Affiliations:** 1 State Key Laboratory of Discovery and Utilization of Functional Components in Traditional Chinese Medicine & School of Pharmaceutical Sciences, Guizhou Medical University, Guiyang, 561113, China; 2 Center of Excellence in Fungal Research, Mae Fah Luang University, Chiang Rai 57100, Thailand; 3 School of Science, Mae Fah Luang University, Chiang Rai, 57100, Thailand; 4 Zest Lanka International, Polonnaruwa, Sri Lanka; 5 Department of Plant Pathology, College of Agriculture, Guizhou University, Guiyang, Guizhou 550025, China

**Keywords:** Holomorphic fungi, Hypocreales, lignocellulolytic fungi, one new genus, saprobes

## Abstract

While exploring the leaf litter fungal diversity in Doi Inthanon National Park, Northern Thailand, we discovered a previously unknown lineage within Niessliaceae. *Muciflexusinthanonensis***gen. et sp. nov.** is therefore introduced to accommodate this new lineage. The link between the sexual and asexual morphs of *Ochronectriathailandica* is also established, while the generic description of *Ochronectria* is updated. A polyphasic approach was employed, consisting of multi-locus phylogenetic analysis using ITS, LSU, *rpb2*, and *tef1-α* along with the morphological evidence to support the findings. *Muciflexusinthanonensis* resembles the spore morphology of *Phialoseptomonium* even though they are phylogenetically distant. *Muciflexusinthanonensis* forms a sister clade with *Trichonectriasetadpressa*, characterised by a long branch, but differs in asexual morphology, possessing clusters of simple or branched conidiophores compared to the sporodochia-like structure of *T.setadpressa*. The discovery of the asexual morph of *Ochronectriathailandica* from a terrestrial habitat confirms the versatile nature of the fungus, which inhabits both aquatic and terrestrial environments. Here we establish the link between the sexual and asexual morphs of *Ochronectria* by revealing the holomorphic nature of *O.thailandica*. This study highlights the diversity of leaf litter fungi and the potential of discovering novel fungal species in northern Thailand.

## ﻿Introduction

Forest ecosystems are one of the most efficient ecosystems in terms of nutrient utilisation, exhibiting the fastest nutrient recycling through a combination of biological and chemical processes (Likens 2013; Niego et al. 2023). This enables forest ecosystems to meet the substantial nutrient demand necessary for sustaining health and functioning (Du et al. 2024). Although some bacteria can decompose plant litter, fungi play a key role in decomposing and nutrient recycling in the lignocellulose matrix (Kjøller et al. 1982; Osono 2007; Wood et al. 2009). Forest trees produce a large amount of leaf litter rich in lignocellulosic compounds. Fungi decompose up to 90% of lignocellulose compounds (Osono 2007), efficiently releasing nutrients into a mobile state, where they become readily available for absorption (Bucher et al. 2004). Additionally, plants establish symbiotic relationships with nitrogen-fixing rhizobia as an adaptation to nutrient limitations (Hyde et al. 2018). However, leaf litter is one of the sources that returns the absorbed nutrients by trees back to the soil, and saprobic fungi play a crucial role in releasing the trapped nutrients back into the soil (Bucher et al. 2004). Thus, identifying the fungi involved in litter decomposition will give insights into nutrient cycling, assess ecosystem resilience, carbon sequestration, and soil fertility, and help to realise the global value of fungi (Hyde et al. 2018).

Hyde and coworkers have been exploring the diversity of micro- and macro-fungi in northern Thailand for almost two decades; more than 500 fungi have been reported from this region by 2018 (Hyde et al. 2018). However, the continued exploration and discoveries have resulted in the number increasing since then (Thitla et al. 2022; Silva et al. 2023; Louangphan et al. 2024). The novelty of microfungi is not as high as that of macrofungi due to less exploration (Hyde et al. 2018). This makes northern Thailand a promising location for exploring microfungi, as many more species are yet to be discovered (Hyde et al. 2024).

Doi Inthanon National Park is a conserved area in northern Thailand representing a mountainous region of Chiang Mai Province (Teejuntuk et al. 2003). The changing geographical features and altitudes create diverse microenvironments that support various organisms and their associated diversity, including microfungi. This makes it a promising location for sheltering diverse species with different micro requirements. Identifying new fungal taxa is important as it expands the horizons of available possibilities and provides more opportunities in fields such as medicine, biochemistry, agriculture, and the function of natural phenomena in the environment (Hyde et al. 2018, 2019, 2024). Identifying novel lineages is crucial for advancing existing taxonomic knowledge and resolving uncertain taxonomic placements. Hence, discovering the missing lineages might serve as a fundamental approach to resolving such taxa. Exploring less-studied environments holds immense potential for discovering previously unknown species. Within the Hypocreales, despite being a well-established taxon, ongoing research continues to reveal new lineages, underscoring the unexplored biodiversity yet to be discovered (Hou et al. 2023; Perera et al. 2023; Sun et al. 2023).

Hypocreales can be found globally in various biotrophic, hemibiotrophic, saprobic, or hypersaprobic habitats (Lombard et al. 2015; Perera et al. 2023). This order is recognised for hosting many fungi that are important in agriculture and medicine (Rossman 1996; Lombard et al. 2015). According to the current update, 29 families are accepted in Hypocreales (Hyde et al. 2024). The members of Hypocreales exhibit a remarkable diversity in lifestyles and habitats (Perera et al. 2023). Hypocreales are generally composed of members with perithecial ascomata, while some genera are cleistothecial (Rossman et al. 1999; Perera et al. 2023). Hypocreales are characterised by transparent, white, pale, bright, or darkly coloured, KOH ±, LA ±, soft, fleshy, or tough ascomata. They can be found superficially on the substrate or embedded within it, sometimes positioned in a stroma that ranges from weak to well-developed. Asci are unitunicate with 2–8 spores. The ascospores range from aseptate to having multiple septa, and they can sometimes be muriform. These ascospores can remain whole or disarticulate. The asexual morph of Hypocreales is usually hyphomycetous, rarely ceolomycetous (Rossman et al. 1999; Hyde et al. 2020; Perera et al. 2023).

During our ongoing exploration of fungal diversity in Doi Inthanon National Park, a comprehensive study on leaf litter led us to discover a previously undocumented genus-level lineage along with the asexual morph of *Ochronectriathailandica*. Here we amend the *Ochronectria* description by including the asexual morph characteristics with the available sexual morph characteristics. This paper provides a detailed account of their morpho-molecular characteristics while presenting the novelty of these findings. These findings provide insights into the lignicolous fungal community in northern Thailand and establish the holomorphic nature of the *Ochronectriathailandica*.

## ﻿Materials and methods

### ﻿Collection, morphological observation, and isolation

Leaf litter that had fallen onto the ground was collected from Doi Inthanon National Park, located in the Chiang Mai District of northern Thailand. The collection information was recorded (Rathnayaka et al. 2024), and the samples were taken to the laboratory at the Centre of Excellence in Fungal Research in paper bags. As detailed by Chomnunti et al. (2014), the leaf samples were incubated to stimulate additional sporulation. Subsequently, a morphological examination was conducted, followed by the isolation of single spores using a spore suspension technique (Senanayake et al. 2020). Spores were isolated into Potato Dextrose Agar (PDA) and Malt Extract Agar (MEA) media plates. The morphological examination of host samples was conducted using a Leica eZ4 educational stereo microscope (Leica, Wetzlar, Germany) and a Nikon EClIPSE Ni compound microscope (Nikon, Tokyo, Japan). Images were captured using the Nikon dS-ri2 digital camera. Measurements were done using the Tarosoft (R) Image Frame Work Program V.09. Adobe Photoshop CS6 Extended software (Adobe Systems, USA) was used to process and present the resulting images. Herbarium materials and live cultures were deposited at the Mae Fah Laung University Herbarium (MFLU) and Mae Fah Luang University Culture Collection (MFLUCC), respectively.

### ﻿DNA extraction and polymerase chain reaction (PCR) amplification

DNA was extracted using the E.Z.N.A.® tissue DNA Kit. The manufacturer’s instructions were followed. Young cultures were used when they were around 1–2 months old. Approximately 30 mg of mycelia was used as the starting material for the DNA extraction.

Four loci, internal transcribed spacer regions (ITS), large subunit rRNA gene (LSU), DNA-directed RNA polymerase II subunit two gene (*rpb2*), and translation elongation factor 1-alpha gene (tef–1α) were amplified using PCR. The primers used and PCR conditions are listed in Table 1. Amplification was performed in a total reaction volume of 25 µl, consisting of 1 µl of genomic DNA template, 1 µl of each forward and reverse primer at a concentration of 20 µm, and 9.5 µl of double-distilled, deionised water, with 12.5 µl of 2× GoTaq® Green Master Mix (PROMEGA, USA). Sequencing was performed by the SolGent Co., Ltd. (South Korea).

**Table 1.** Loci, primers, and PCR conditions used in this study.

Loci	PCR Primers	Sequence (5′–3′)	PCR Cycles	References
ITS	ITS5	GGA AGT AAA AGT CGT AAC AAG G	(95 °C: 30 s, 55 °C: 50 s, 72 °C: 1 min) × 35 cycles	White et al. (1990)
	ITS4	TCC TCC GCT TAT TGA TAT GC		
LSU	LR0R	GTA CCC GCT GAA CTT AAG C	(95 °C: 30 s, 52 °C: 30 s, 72 °C: 1 min) × 35 cycles	Rehner and Samuels (1994); Vilgalys and Hester (1990)
	LR5	TCC TGA GGG AAA CTT CG		
*tef1*–*α*	EF1–983F	GCY CCY GGH CAY CGT GAY TTY AT	(95 °C: 30 s, 55 °C: 50 s, 72 °C: 1 min) × 35 cycles	Rehner and Buckley (2005)
	EF1–2218R	AT GAC ACC RAC RGC RAC RGT YTG		
*rpb2*	RPB25F2	GGG GWG AYC AGA AGA AGGC	(95 °C: 1 min, 52 °C: 30 s, 72 °C: 2 min) × 35 cycles	Sung et al. (2007)
	RPB27CR	CCC ATR GCT TGY TTR CCC AT		

### ﻿Phylogenetic analyses

Forward and reverse sequences were assembled using the Staden Package (Staden et al. 2003), and the resulting sequences were compared against the NCBI GenBank database (Sayers et al. 2020). Related reference sequences were downloaded from the GenBank database (Suppl. material 1). Individual data sets referring to each gene region were aligned using MAFFT version 7 with the --auto flag (Kuraku et al. 2013; Katoh et al. 2019). The sequences were trimmed automatically using trimAl 1.2rev57 with the -*gt* (0.5) option (Capella-Gutiérrez et al. 2009). Sequence Matrix was used to concatenate the alignments in the order of ITS, LSU, *rpb2*, and *tef1*–*α* (Vaidya et al. 2011). The best-fit model for each individual dataset was selected using jModelTest2 (Guindon and Gascuel 2003; Darriba et al. 2012). Concatenated data sets were analysed using Maximum Likelihood (ML) and Bayesian Inference (BI) analyses.

The Maximum Likelihood tree was generated using the IQ-Tree web server, available at http://iqtree.cibiv.univie.ac.at/ (Trifinopoulos et al. 2016). Bayesian inference was performed on the CIPRES Science Gateway platform using the MrBayes 3.2 tool (Huelsenbeck and Ronquist 2001; Ronquist and Huelsenbeck 2003; Ronquist et al. 2012). It was performed by four simultaneous Markov Chain Monte Carlo (MCMC) chains in two runs. Each run consisted of ten million generations, and trees were sampled every 1,000 generations. The first and final 25% results were discarded as the burn-in. The rest was used to calculate the BI posterior probabilities (PP). The final consensus phylograms were visualised using the FigTree drawing tool (version 1.4.0, Rambaut 2012) and edited using Microsoft PowerPoint. The guidelines of Maharachchikumbura et al. 2021 were used for introducing new taxa.

Pairwise evolutionary divergence between sequences was estimated using MEGA 12 (Kumar et al. 2024). Analyses were conducted using the Kimura 2-parameter model, with rate variation among sites modelled using a gamma distribution (shape parameter = 1). The partial deletion option was applied to eliminate all positions with less than 95% site coverage (Kimura 1980).

## ﻿Results

### ﻿ Muciflexus

Taxon classificationFungiHypocrealesNiessliaceae

V. S. Silva & Jayaward. gen. nov.

AA95E355-D1AE-5568-B5BA-EC254AC29DBD

Index Fungorum: IF903024

Facesoffungi Number: FoF17005

#### Classification.

Niessliaceae, Hypocreales, Hypocreomycetidae, Sordariomycetes.

#### Etymology.

”Muci-” refers to the slimy nature of the conidial masses, and “flexus” refers to the flexibility or branching of the conidiophores.

#### Description.

Surface mycelium composed of hyaline, smooth-walled, branched hyphae. Conidiophores arising directly from hyphae, straight to flexuous, erect, branched, hyaline, arranged in dense clusters or solitarily, cylindrical and slightly tapering towards the apical end. Conidiogenous cells integrated, adhering in slimy masses, apically produce conidia. Conidia often aseptate or multiseptate, granular, fusoid, apex obtuse, base truncate, straight to slightly curved, hyaline, smooth-walled.

#### Type species.

*Muciflexusinthanonensis* V. S. Silva, K.D. Hyde & Jayaward.

### ﻿ Muciflexus inthanonensis

Taxon classificationFungiHypocrealesNiessliaceae

V. S. Silva, K.D. Hyde & Jayaward. sp. nov.

A6A09E30-EFA3-5A6F-9430-BBE102E811B8

Index Fungorum: IF903018

Facesoffungi Number: FoF17006

Fig. 1

#### Holotype.

MFLU 24–0382.

**Figure 1.**
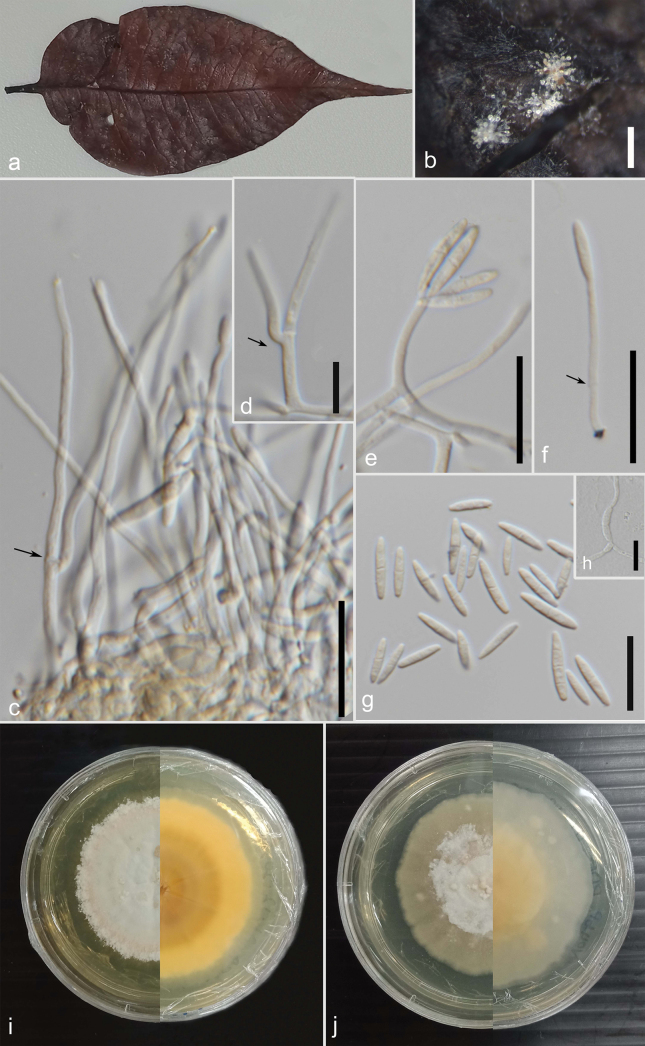
*Muciflexusinthanonensis* (Holotype MFLU24–0382) **a** host **b** a closer view of the colonies on the host substrate **c–f** conidiophores with attached conidia (arrows **c–d** branched conidiophore, **f** basal septation of conidiophore) **g** conidia **h** germinated spore **i** front and the reverse view of the culture on MEA media **j** front and the reverse view of the culture on PDA media. Scale bars: 200 µm (**b**); 20 µm (**c, e, f**); 10 µm (**d, g, h**).

#### Etymology.

”inthanonensis” refers to the type locality, Doi Inthanon, Thailand.

#### Description.

Saprobic on a leaf of a fallen unknown broadleaf species. Sexual morph: Undetermined. Asexual morph: Hyphomycetous, erect, appear in solitary or in groups. Surface mycelium composed of branched, hyaline, smooth-walled hyphae. Conidiophores erect, simple or occasionally branched, straight to flexuous, arranged in dense clusters or solitary, arising directly from hyphae, with 0–1 basal septa, cylindrical, slightly tapering towards the apical end, hyaline, smooth-walled, 19.5–85 (x̄ = 55.3, n = 10) µm, base 2.2–3.2 (x̄ = 2.6, n = 10) µm, apex 1.3–1.9 (x̄ = 1.5, n = 10) µm. Conidiogenous cells integrated, apically produce 1–4 conidia, adhering in slimy masses. Conidia solitary or occasionally grouped, straight to slightly curved, often aseptate or 1-septate or rarely 2–3 septate, granular, fusoid, apex obtuse, base truncate, hyaline, smooth-walled, 10.5–19 (x̄ = 13.9, n = 68) × 2.1–3.3 (x̄ = 2.7, n = 68) µm, L/W 5.14.

#### Culture characteristics.

In both PDA and MEA media, culture diameter reaches an average of 6 mm within 5 days. On both media, colonies are flat. After about 45 days on the PDA media in the front, it develops in the buff with sparse white aerial mycelium at the centre. On the reverse also, it develops into buff with a smooth, entire margin. On the MEA media in front view, it develops into sparse mycelium white at the centre with an orangish margin ring extending to white aerial mycelium. On the reverse, it is buff and concentric rings gradually becoming light.

#### Material examined.

Thailand • Chiang Mai Province, Doi Inthanon National Park, on a fallen unidentified broadleaf species, 30 November 2022, V. S. Hittanadurage Silva, V046 (holotype MFLU 24–0382); ex-type living culture (MFULCC 24–0502).

#### GenBank accession numbers.

ITS: PQ528132, LSU: PQ528133, SSU: PQ528134, *rpb2*: PQ590309, *tef1*–*α*: PQ568247.

**Figure 2.**
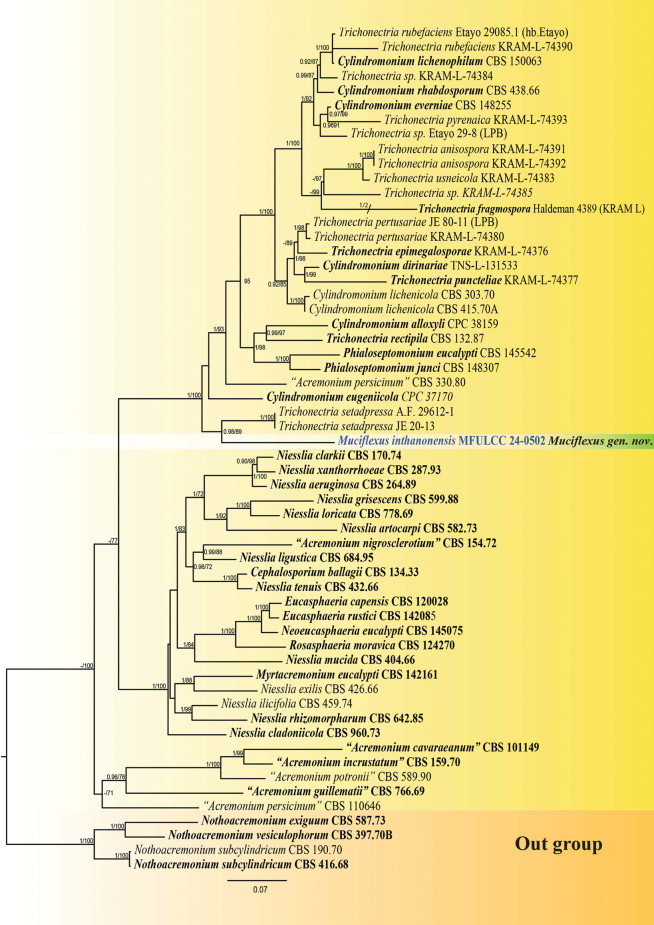
Maximum likelihood phylogenetic tree generated from ITS, LSU, *rpb2*, and *tef1*–*α* sequence data of selected taxa from Niessliaceae. The tree is rooted with four taxa from Nothoacremoniaceae (CBS 416.68, CBS 190.70, CBS 587.73, and CBS 397.70B). The newly generated sequence is in bold blue. Holotype and ex-type strains are in bold text. Bayesian posterior probabilities (BYPP) ≥ 0.95 and maximum likelihood bootstrap (MLBS) values ≥ 70% are shown at the nodes.

In the phylogenetic tree, the dataset comprised 58 strains representing Niessliaceae, including *incertae sedis* taxa. Following Hou et al. (2023), taxa with the species name, which are not yet formally accepted or validated under the International Code of Nomenclature for fungi, were also included. They are represented within “”. The outgroup is represented by four taxa from Nothoacremoniaceae (CBS 416.68, CBS 190.70, CBS 587.73, and CBS 397.70). The final concatenated nucleotide alignment was composed of ITS, LSU, *rpb2*, and *tef1*–*α* with 2974 sites in total (ITS = 1–569; LSU = 570–1410; *rpb2* = 1411–2166; *tef1*–*α* = 2167–2974). The maximum likelihood and Bayesian analyses yielded similar tree topologies, which are combined in Fig. 2. The maximum likelihood tree default setting in the IQ-TREE web server was used, and for the BI tree, the combined region run quality was checked using Tracer v1.7.2 after the runs were completed. All runs were conducted with effective sample size (ESS) values for all parameters. The alignment contained 1,340 unique sites (ITS = 357; LSU = 273; *rpb2* = 406; *tef1*–*α* = 304).

#### Notes.

Hou et al. (2023) conducted phylogenetic analyses identifying two distinct clades (Clade G & Clade H) within the family Niessliaceae. The authors highlighted that the phylogenetic relationships within Niessliaceae remain unresolved, with several genera, viz., *Niesslia*, *Cylindromonium*, and *Trichonectria*, exhibiting polyphyletic characteristics (Hou et al. 2023).

In our analysis, *Muciflexusinthanonensis* clustered within H. Niessliaceae (Hou et al. 2023), alongside taxa from *Cylindromonium*, *Trichonectria*, and *Phialoseptomonium*. However, *Trichonectria* is currently classified as Hypocreales genera *incertae sedis* (Perera et al. 2023; Hyde et al. 2024). *Muciflexusinthanonensis* formed a sister clade to *Trichonectriasetadpressa*, with a BYPP of 0.98 and MLBS of 89% statistical support. The noticeable branch length difference suggests that *M.inthanonensis* forms a distinct lineage, which may be attributed to the inclusion of two additional gene regions (*rpb2* and *tef1–α*) not available for *T.setadpressa* and potential genetic novelty. Morphologically, *T.setadpressa* is characterised by sporodochia-like conidiomata with subglobose to broadly ellipsoidal conidia as its asexual morph (Flakus et al. 2019), whereas *M.inthanonensis* produces clusters of simple or occasionally branched conidiophores with fusoid conidia. Additionally, *Trichonectria* is a lichenicolous genus (Perera et al. 2023), while *M.inthanonensis* is saprobic, found on a fallen unidentified broadleaf species. Furthermore, a pairwise comparison of all gene regions between *M.inthanonensis* and *T.setadpressa* (Table 2) provides further evidence supporting the genetic distinctiveness of the newly introduced genus.

**Table 2.** Pairwise base pair (Bp) comparison of *Muciflexusinthanonensis* to other related taxa.

	*Cylindromoniumeugeniicola* (CPC 37170)		*Trichonectriasetadpressa* (JE 20–13)		*Phialoseptomoniumeucalypti* (CBS 145542)		*Phialoseptomoniumjunci* (CBS 148307)	
	Bp differences	Gaps	Bp differences	Gaps	Bp differences	Gaps	Bp differences	Gaps
**ITS**	96/588(16%)	31/588(5%)	95/573(17%)	36/573(6%)	104/588(18%)	36/588(6%)	99/588(17%)	38/588(6%)
**LSU**	10/414(2%)	0/414(0%)	7/402(2%)	2/402(0%)	15/424(4%)	0/424(0%)	20/408(5%)	3/408(0%)
***tef1*–*α***	66/768(9%)	2/768(0%)	N/A	N/A	81/770(11%)	6/770(0%)	N/A	N/A
***rpb2***	N/A	N/A	N/A	N/A	No significant similarity		N/A	N/A

N/A – Sequence is not available.

Morphologically, *Muciflexusinthanonensis* closely resembles *Phialoseptomonium*, particularly in spore characteristics. Its solitary fusoid conidia, which are granular, hyaline, smooth-walled, and adhere in slimy masses, are similar to those of *Phialoseptomonium* (Crous et al. 2019a). However, *M.inthanonensis* can be distinguished by its aseptate or 1–3-septate, grouped conidia and comparatively smaller spore size (*P.eucalypti*: L/W = 6.7, *P.junci*: L/W = 6.2, *M.inthanonensis*: L/W = 5.14). Phylogenetically, *M.inthanonensis* clusters distantly from *Phialoseptomonium*. While *Phialoseptomonium* species have been reported as saprobes, taxonomic differentiation based on genetic data is also crucial. According to Raja et al. 2017, variation in the LSU gene region is indicative of differences at intermediate taxonomic levels, such as family and genus. Pairwise comparisons between *M.inthanonensis* and *Phialoseptomonium* species (Table 2) further support the likelihood that *M.inthanonensis* does not belong to the same genus.

*Cylindromonium* exhibits polyphyletic behaviour and was established to accommodate *Acremonium*-like taxa characterised by unbranched, hyaline conidiophores and cylindrical conidia (Crous et al. 2019b). Apart from *C.alloxyli* and *C.eugeniicola*, the remaining members of the genus are lichenicolous (Suppl. material 2). *Cylindromoniumalloxyli* is mycophilic on *Meliola* and was found on *Alloxylonpinnatum* leaves (Crous et al. 2020), whereas *C.eugeniicola* is saprobic on leaves (Crous et al. 2019). Crous et al. 2019b mentioned the morphological resemblance between *Cylindromonium* and *Phialoseptomonium*; however, they can be distinguished by the cylindrical conidia of *Cylindromonium*. Based on this, pairwise genetic distances were calculated among all *Cylindromonium* and *Phialoseptomonium* species (Suppl. material 3), with the resulting *p*-distance values presented in Table 3. These values were then compared against the *p*-distance values of *Muciflexusinthanonensis* with both *Cylindromonium* and *Phialoseptomonium*, yielding the following results: ITS: 0.14–0.19, LSU: 0.12–0.06, *tef1–α*: 0.12–0.15, and *rpb2*: 0.31–0.40. These values fall within or exceed the range that differentiates *Cylindromonium* and *Phialoseptomonium*. Additionally, *Muciflexus* can be morphologically distinguished from *Cylindromonium* by its occasionally branched conidiophores and fusoid, occasionally grouped conidia. These combined morphological and phylogenetic differences provide strong support for the novelty of the proposed genus.

**Table 3.** Range of p-distance values of separating *Cylindromonium* from *Phialoseptomonium*.

	p-distance value	
	Maximum	Minimum
**ITS**	0.14	0.09
**LSU**	0.05	0.02
***tef1*–*α***	0.16	0.09
***rpb2***	0.33	0.26

Based on these host associations along with morphological and phylogenetic evidence, it is inconclusive to place *Muciflexusinthanonensis* in any of the genera within the H clade of Niessliaceae (Hou et al. 2023). Therefore, here we propose a new genus, *Muciflexus*, to accommodate *Muciflexusinthanonensis*.

### ﻿ Ochronectria

Taxon classificationFungiHypocrealesBionectriaceae

Rossman & Samuels, emend V. S. Silva & Jayaward.

EEEB06BA-3089-54A9-A619-A37669A44481

Index Fungorum: IF28315

Facesoffungi Number: FoF13003

#### Classification.

Bionectriaceae, Hypocreales, Hypocreomycetidae, Sordariomycetes.

#### Remarks.

*Ochronectria* was established by Rossman et al. (1999), accommodating *Ochronectriacalami* as the type species. The genus features subglobose to globose ascomata that become cupulate upon drying, peridium with three distinct layers, clavate asci, and fusiform ascospores containing guttules (Rossman et al. 1999; Lechat 2010; Li et al. 2016). Three species are accepted under *Ochronectria*, with no report on the asexual morph (Index Fungorum 2025). Discovery of the asexual form of *Ochronectriathailandica* in this study reveals the asexual morph of *Ochronectria*. Therefore, the genus description is emended here with general asexual morphology.

#### Description.

**Sexual morph**: as described by the original description, Rossman et al. (1999)

**Asexual morph**: Hyphomycetous. Colonies on the host are solitary to gregarious, and the vegetative mycelium is superficial. Conidiophores erect, mononemotous, unbranched, 2-septate, hyaline, smooth-walled. Conidiogenous cells elongate, hyaline, holoblastic, and apically produce monoblasitic conidia. Conidia aseptate, ellipsoidal to cylindrical, rarely ovoid, hyaline, smooth-walled.

### ﻿ Ochronectria thailandica

Taxon classificationFungiHypocrealesBionectriaceae

Q.J. Shang & K.D. Hyde

47DC37B5-99A1-5661-AF9E-1F65CC973340

Index Fungorum: IF551918

Facesoffungi Number: FoF01815

Fig. 3

#### Description.

**Sexual morph**: see Li et al. (2016). **Asexual morph**: saprobic and hyphomycetous. Colonies on the host solitary to gregarious, vegetative mycelium superficial. Conidiophores erect, mononematous, smooth, hyaline, unbranched, 2-septate, 70–89(x̄ = 77, n = 9) µm. Conidiogenous cell elongated, hyaline, holoblastic, apically producing monoblastic conidia, 22–37 (x̄ = 28, n = 9) µm. Conidia hyaline, smooth-walled, aseptate, ellipsoidal to cylindrical rarely ovoid, 4.5–8.5 × 2–3 (x̄ = 6–2.5, n = 40) µm, L/W 2.5.

**Figure 3.**
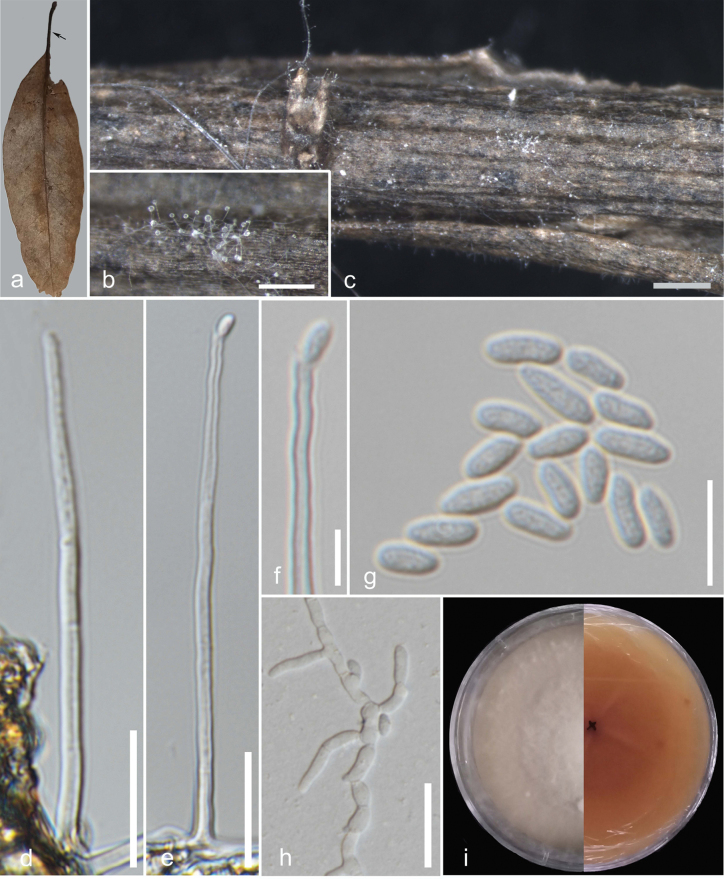
*Ochronectriathailandica* (MFLU 24–0383) **a** host (arrow points to the colonies on the petiole) **b, c** a closer view of the colonies on the host substrate **d–f** conidiophores with attached conidia **g** conidia **h** germinated spore **i** front and the reverse view of the culture on MEA media. Scale bars: 200 µm (**b**); 500 µm (**c**); 20 µm (**d–e, h**); 5 µm (**f**); 10 µm (**g**).

#### Culture characteristics.

Conidia germinating on MEA within 24 hours. Colonies on MEA reaching 1.4 mm diam. within 6 days in the dark at 25 °C, edge entire, flat or effuse, sparse. After 7 days colonies become white on the front face, and from below, reddish yellow gradually becomes slightly dark when mature.

#### Material examined.

Thailand • Chiang Mai Province, Doi Inthanon National Park, on a petiole of a fallen broadleaf species, 30 November 2022, Veenavee Silva, V054a, MFLU 24–0383, MFULCC 24–0503

#### GenBank accession numbers.

ITS: PQ454717, LSU: PQ454721

**Figure 4.**
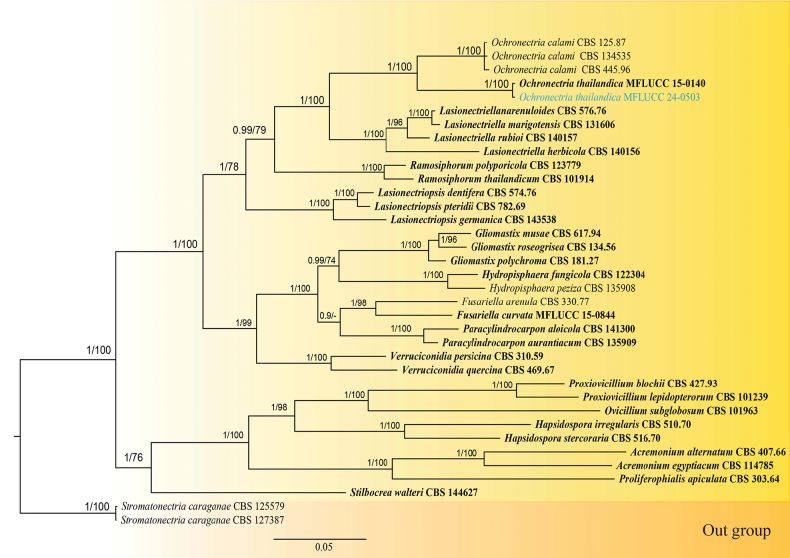
Bayesian inference phylogenetic tree generated from ITS, LSU, *rpb2*, and *tef1*–*α* sequence data for selected taxa from Bionectriaceae. The tree is rooted with two taxa from Stromatonectriaceae (CBS 125579 and CBS 127387). The newly generated sequence is in blue. Holotype and ex-type strains are in bold text. BYPP ≥ 0.95 and MLBS values ≥ 70% are shown at the nodes.

In the phylogenetic analysis, the dataset consisted of 36 strains from Bionectriaceae, with two taxa from Stromatonectriaceae (CBS 125579 and CBS 127387) serving as the outgroup. The final concatenated nucleotide alignment included ITS, LSU, *rpb2*, and *tef1-α* sequences, totalling 2854 sites (ITS: 1–514; LSU: 515–1290; *rpb2*: 1291–2046; *tef1*–*α*: 2047–2854). Both maximum likelihood and Bayesian analyses produced similar tree topologies; they were combined in Fig. 4, using the BI tree as the base. The IQ-TREE web server’s default settings were used for the maximum likelihood tree. At the same time, Tracer v1.7.2 was employed to assess the run quality of the BI tree, ensuring effective sampling size (ESS) values for all parameters. The alignment contained 1,096 unique sites (ITS: 262; LSU: 145; *rpb2*: 419; *tef1*–*α*: 270). Although the target species, *Ochronectriathailandica*, only had ITS and LSU sequences, all four loci were used in the multi-loci phylogenetic analysis, resulting in a stable tree.

#### Notes.

*Ochronectriathailandica* was introduced by Li et al. (2016) in Chiang Rai Province, Thailand, from unidentified wood in the water. Our collection from Doi Inthanon includes an isolate found on the petiole of a fallen broadleaf species, which is an asexual morph. In the multi-locus phylogenetic analysis, this isolate clusters with the ex-type strain of *Ochronectriathailandica* (MFLUCC 15-0140) with BYPP of 1 and MLBS of 100% support. Based on base pair comparisons, the ITS region is identical (99%), with two gaps, and the LSU region is also identical (100%), with no gaps. This confirms that our isolate shares the same identity as the type strain of *Ochronectriathailandica*. As a result, we introduce the asexual morph of *O.thailandica* here, supported by graphical illustrations and morpho-phylogenetic evidence. Previously, *Ochronectria* was recognised as a genus with only a sexual morph, but our discovery establishes its holomorphic nature. Thus, we amend the genus description to include the asexual morph.

## ﻿Discussion

This study introduces the new genus *Muciflexus* with *Muciflexusinthanonensis* sp. nov. and the asexual morph of *Ochronectriathailandica* based on a polyphasic approach, discovered during our ongoing exploration of fungal diversity in Doi Inthanon National Park.

The phylogeny of Niessliaceae remains unresolved (Hou et al. 2023), comprising several polyphyletic genera, such as *Niesslia*, *Cylindromonium*, and *Trichonectria*. The discovery of *Muciflexus* reveals a previously unknown lineage within Niessliaceae. Identifying new lineages like this is crucial for resolving unresolved taxonomic placements, as they may provide missing information needed for clarity. Previously, *Ochronectria* was known only for its sexual morph. The discovery of the asexual morph of *Ochronectriathailandica* extends our understanding of the genus by confirming its holomorphic nature. As a result, the genus description is amended to include the asexual morph characteristics. According to Species Fungorum (2025), *Ochronectria* includes three accepted species: *O.thailandica*, *O.calami*, and *O.courtecuissei*, which have been reported in Asia (Indonesia and Thailand) and Europe (France) across both terrestrial and freshwater environments (Rossman et al. 1999; Lechat 2010; Li et al. 2016). The sexual morph of *O.thailandica* was previously identified on decaying wood in freshwater habitats; in contrast, our study reports the asexual morph on a decaying dicotyledon leaf petiole in a terrestrial habitat (Li et al. 2016). This highlights the ecological versatility of *O.thailandica*, suggesting it can inhabit both freshwater and terrestrial environments. Consequently, *O.calami* and *O.courtecuissei* may also possess this habitat adaptability.

These findings provide deeper insight into the leaf litter fungal diversity of northern Thailand, confirming its potential to harbour more undiscovered species. The fungi were found in saprobic life forms, highlighting their role in nutrient recycling within the forest ecosystem. Additionally, revealing the versatile nature of *Ochronectriathailandica* to thrive in both aquatic and terrestrial environments further confirms the contribution of fungi to nutrient cycling in both ecosystems.

## Supplementary Material

XML Treatment for Muciflexus

XML Treatment for Muciflexus inthanonensis

XML Treatment for Ochronectria

XML Treatment for Ochronectria thailandica

## ﻿Additional information

### Author ORCIDs

Veenavee S. Hittanadurage Silva https://orcid.org/0000-0001-8921-1370

Ruvishika S. Jayawardena https://orcid.org/0000-0001-7702-4885

Rekhani H. Perera https://orcid.org/0000-0002-1360-5362

Qirui Li https://orcid.org/0000-0001-8735-2890

Kevin D. Hyde https://orcid.org/0000-0002-2191-0762

### Data availability

All of the data that support the findings of this study are available in the main text or Supplementary Information.

## Supplementary materials

Supplementary material 1 Niessliaceae and Bionectriaceae taxa table Data type xlsx https://binary.pensoft.net/file/1322506This dataset is made available under the Open Database License (http://opendatacommons.org/licenses/odbl/1.0/). The Open Database License (ODbL) is a license agreement intended to allow users to freely share, modify, and use this Dataset while maintaining this same freedom for others, provided that the original source and author(s) are credited.Veenavee S. Hittanadurage Silva, Ruvishika S. Jayawardena, Rekhani H. Perera, Qirui Li, Kevin D. Hyde

Supplementary material 2 Asexual morph comparison Data type xlsx https://binary.pensoft.net/file/1322507This dataset is made available under the Open Database License (http://opendatacommons.org/licenses/odbl/1.0/). The Open Database License (ODbL) is a license agreement intended to allow users to freely share, modify, and use this Dataset while maintaining this same freedom for others, provided that the original source and author(s) are credited.Veenavee S. Hittanadurage Silva, Ruvishika S. Jayawardena, Rekhani H. Perera, Qirui Li, Kevin D. Hyde

Supplementary material 3 Pairwise distance calculation of *Cylindromonium* and *Phialoseptomonium* Data type xlsx https://binary.pensoft.net/file/1322508This dataset is made available under the Open Database License (http://opendatacommons.org/licenses/odbl/1.0/). The Open Database License (ODbL) is a license agreement intended to allow users to freely share, modify, and use this Dataset while maintaining this same freedom for others, provided that the original source and author(s) are credited.Veenavee S. Hittanadurage Silva, Ruvishika S. Jayawardena, Rekhani H. Perera, Qirui Li, Kevin D. Hyde
